# Dynamic Ultrasonography for Imaging Pediatric Fat Pad Herniation through the Lateral Patellar Retinaculum

**DOI:** 10.3390/diagnostics12102523

**Published:** 2022-10-17

**Authors:** Wei-Ting Wu, Ke-Vin Chang, Kuan-Wen Wu, Kamal Mezian, Vincenzo Ricci, Levent Özçakar

**Affiliations:** 1 Department of Physical Medicine and Rehabilitation, National Taiwan University Hospital, Bei-Hu Branch, Taipei 10845, Taiwan; 2 Department of Physical Medicine and Rehabilitation, College of Medicine, National Taiwan University, Taipei 10048, Taiwan; 3 Center for Regional Anesthesia and Pain Medicine, Wang-Fang Hospital, Taipei Medical University, Taipei 11600, Taiwan; 4 Department of Orthopaedic Surgery, National Taiwan University Hospital, Taipei 10048, Taiwan; 5 Department of Rehabilitation Medicine, Charles University, First Faculty of Medicine and General University Hospital in Prague, 12800 Prague, Czech Republic; 6 Physical and Rehabilitation Medicine Unit, Luigi Sacco University Hospital, ASST Fatebenefratelli-Sacco, 20157 Milan, Italy; 7 Department of Physical and Rehabilitation Medicine, Hacettepe University Medical School, Ankara 06100, Turkey

**Keywords:** knee, child, sonography, fat pad, diagnostic imaging

## Abstract

A 3-year-old boy was found to have a painless mass over his right anterior lateral knee for the previous 6 months. The mass was hardly identified during knee extension and became visible upon squatting. There was no specific finding on ultrasound imaging over his right knee at the supine extended position. During squatting, ultrasound imaging showed an ill-demarcated hyperechoic mass protruding next to the cartilage overlying the distal femur towards the subcutaneous layer through a slit over the lateral patellar retinaculum. Herniation of the fat pad through a defect in the lateral patellar retinaculum was diagnosed. Our case highlights the usefulness of ultrasound examination as regards the lateral patellar retinaculum defect in pediatric knees, as well as its capability for dynamic scanning to capture the exact “pathological moment”.

A 3-year-old boy was found to have a painless mass over his right anterior lateral knee. The onset of the mass was about 6 months prior to examination. Besides the mass, there was no obvious swelling at other compartments of his right knee. His mother did not recall any trauma in the affected knee. The mass was hardly identified during knee extension and became visible upon squatting. The mass was soft and elastic. No associated skin-color change was visualized. He was brought to the pediatric orthopedic clinic, where physical examination revealed normal bony alignment of his knees. Based on the initial impression of a ganglion cyst, he was referred for an ultrasound check-up. All of the sonographic images presented in this case report were obtained using a 13–18 MHz high-frequency linear transducer (Aplio 500, Canon Medical Systems Europe B.V., Zoetermeer, The Netherlands). The scanning depth was set between 2 and 3 cm, with the focus fixed at 1 cm deep.

With his right knee extended in the supine position, ultrasound imaging over the suprapatellar region demonstrated no effusion, an intact quadriceps tendon, a non-ossified patella, prominent femoral cartilage, and a clear epiphyseal growth plate (Figure 1A). The transducer was relocated distally to scan the infrapatellar area. Likewise, the fibrillar appearance of the patellar tendon remained without any remarkable pathologies (Figure 1B).

As no obvious mass was identified in the supine position, we then asked him to squat so as to make the mass more visible (Figure 2).

Ultrasound imaging showed an ill-demarcated hyperechoic mass protruding next to the cartilage overlying the distal femur towards the subcutaneous layer through a slit over the lateral patellar retinaculum (Figure 3A and Video S1). No increased vascularity was visualized inside or surrounding the lesion (Figure 3B). Repositioning the knee back to extension while lying supine, the mass was seen sinking to the space underneath the lateral patellar retinaculum (Figure 3C). Accordingly, herniation of the fat pad through a defect in the lateral patellar retinaculum was diagnosed.

Concerning musculoskeletal sonography, substantial differences might exist between children and adults [1,2]. Compared with those of adults, children’s bones harbor growth plates, profound cartilage, and visible juxta-articular vascularity [3]. At the knee region in a 3-year-old child, the non-ossified patella appeared anechoic, mimicking effusion or a ganglion cyst. Furthermore, the growth plates manifest as discontinuity over the end of long bones, which can be misrecognized as fractures [4]. All of the aforementioned factors might lead to difficulty in interpreting pediatric knee ultrasound examinations. In children with unilateral signs/symptoms, comparison with the asymptomatic side would be helpful in determining whether the finding is normal or pathological.

The anterior lateral knee component is a multilayer structure, whereas a two-layered or a three-layered model has been proposed by previous studies [5,6]. A recent study investigating the anatomical, histological, and radiological aspects of the anterior lateral knee compartment revealed that the lateral patellar retinaculum could actually be differentiated from the lateral patellar femoral ligament [7]. A focal defect over the lateral patellar femoral retinaculum is not rare and is mostly asymptomatic. One magnetic resonance imaging study on adult patients revealed that the defect was associated with a decrease in the infrapatellar fat pad thickness, but not a lower Kellgren–Lawrence radiological grade of the knee joint [8]. The authors also found that the correlation of the defect size with age, body weight, height, subcutaneous fat thickness, or the infrapatellar fat pad area was not statistically significant. Another ultrasound study on 48 healthy adult volunteers demonstrated that vessels could be consistently identified lateral to the patellar tendon, penetrating the defect to reach to the subcutaneous layer [9]. The aforementioned research also revealed that the mean transverse diameter of the defect was 2.75 mm. Compared with magnetic resonance imaging, dynamic examination is more easily conducted under ultrasound [10]. A case report of a 45-year-old man demonstrated that herniation of the fat pad through a slit of the lateral patellar retinaculum could be provoked after knee flexion and readily identified with ultrasound imaging [11]. The defect in the above-discussed case might be derived from the anterolateral portal during the antecedent arthroscopic surgery. Similarly, the herniated fat pad in our patient could only be seen during squatting. To this end, our case highlighted the usefulness of ultrasound examination as regards pediatric knee problems, as well as its capability for dynamic scanning to capture the exact “pathological moment”.

## Figures and Tables

**Figure 1:**
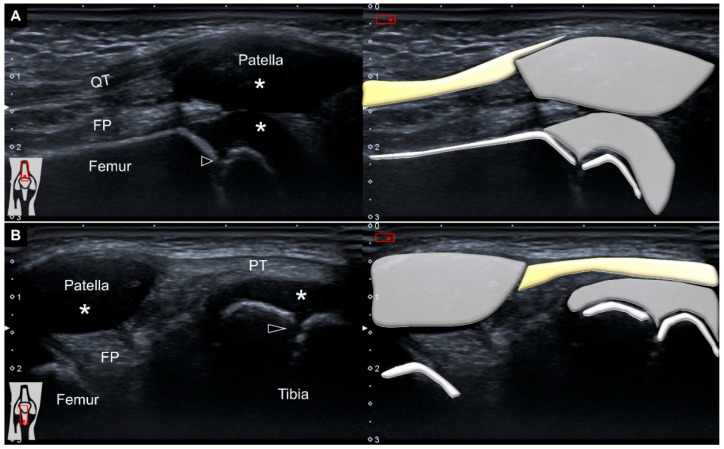
Ultrasound imaging and schematic drawing of the (**A**) suprapatellar and (**B**) infrapatellar regions. QT: quadriceps tendon; FP: fat pad; PT: patellar tendon; *: hyaline cartilage; black arrowheads: growth plate.

**Figure 2:**
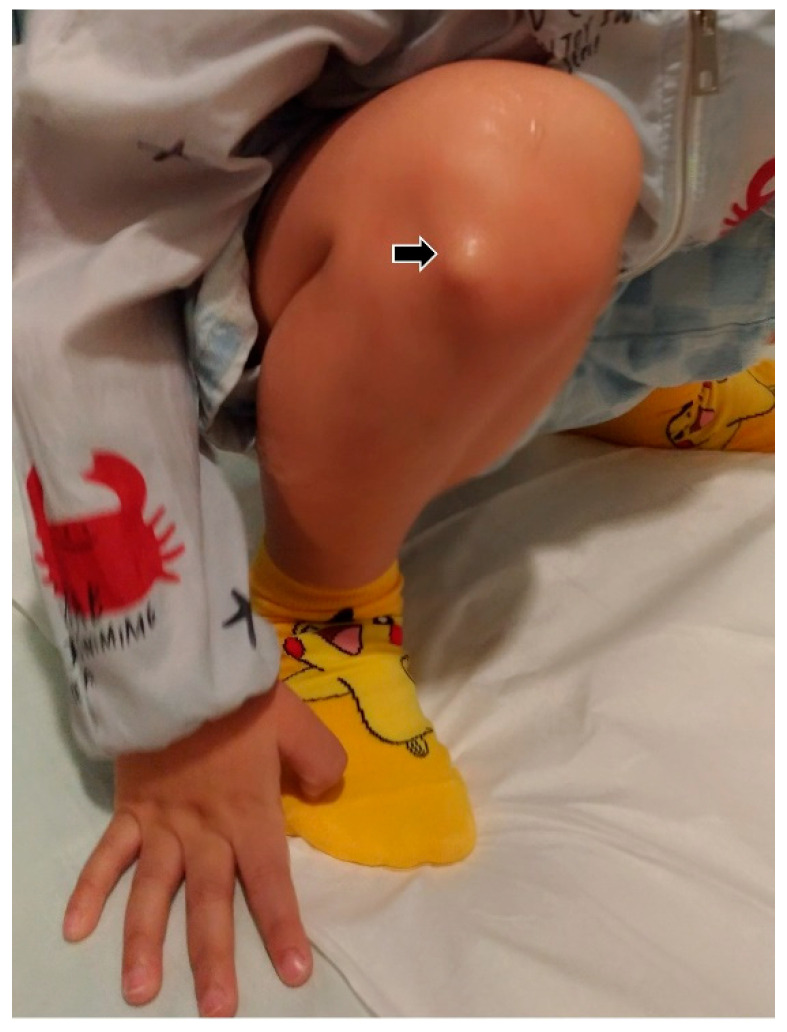
A mass (black arrow) was seen protruding from the lateral infrapatellar region when squatting.

**Figure 3:**
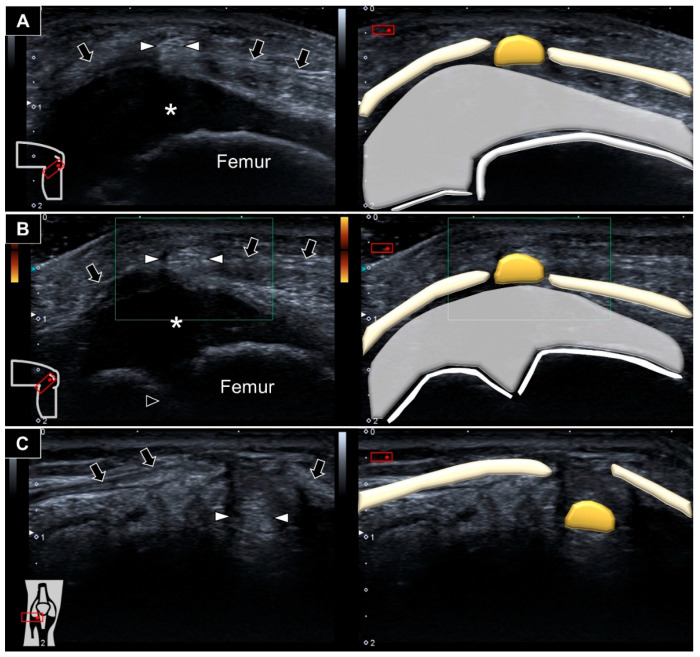
Hyperechoic fat pad (white arrowheads) was seen protruding through a defect in the lateral patellar retinaculum (black arrows) (**A**). There was no vascularity surrounding or inside the protruded fat pad during squatting (**B**). The fat pad was visualized sinking underneath the lateral retinaculum upon knee extension (**C**). *: hyaline cartilage.

## Data Availability

Data are contained within the main text of the manuscript.

## Supplementary Materials

The following supporting information can be downloaded at: https://www.mdpi.com/article/10.3390/diagnostics12102523/s1: Video S1: Dynamic ultrasound examination for the defect in the lateral patellar retinaculum.

## References

[1] Chang K.V., Sahin Onat S., Lee C.W., Kara M., Hung C.Y., Ozcakar L. (2016). EURO-MUSCULUS/USPRM basic scanning protocols revisited in children. Eur. J. Phys. Rehabil. Med..

[2] Ozcakar L., Kara M., Chang K.V., Tok F., Hung C.Y., Akkaya N., Wu C.H., Carli A.B., Hsiao M.Y., Tekin L. (2015). EURO-MUSCULUS/USPRM. Basic scanning protocols for knee. Eur. J. Phys. Rehabil. Med..

[3] Tok F., Demirkaya E., Özçakar L. (2011). Musculoskeletal ultrasound in pediatric rheumatology. Pediatr. Rheumatol..

[4] de Borja C., Watkins R., Woolridge T. (2022). Common Ultrasound Applications for Pediatric Musculoskeletal Conditions. Curr. Rev. Musculoskelet. Med..

[5] Fulkerson J.P., Gossling H.R. (1980). Anatomy of the knee joint lateral retinaculum. Clin. Orthop. Relat. Res..

[6] Merican A.M., Amis A.A. (2008). Anatomy of the lateral retinaculum of the knee. J. Bone Joint Surg. Br..

[7] Biz C., Stecco C., Crimì A., Pirri C., Fosser M., Fede C., Fan C., Ruggieri P., De Caro R. (2022). Are Patellofemoral Ligaments and Retinacula Distinct Structures of the Knee Joint? An Anatomic, Histological and Magnetic Resonance Imaging Study. Int. J. Environ. Res. Public Health.

[8] Kim J.S., Yun S.J., Jin W., Kim G.Y., Park S.Y., Park J.S., Ryu K.N. (2017). A Focal Defect at the Lateral Patellar Retinaculum on Clinical Knee MRI and Cadaveric Study: A Normal Variant or Pathologic Lesion?. AJR Am. J. Roentgenol..

[9] Moraux A., Bianchi S., Tassery F., Le Corroller T. (2019). The lateral patellar retinaculum defect: Anatomical study using ultrasound. Skelet. Radiol..

[10] Wu W.T., Lin C.Y., Shu Y.C., Chen L.R., Ozcakar L., Chang K.V. (2022). Subacromial Motion Metrics in Painful Shoulder Impingement: A Dynamic Quantitative Ultrasound Analysis. Arch. Phys. Med. Rehabil..

[11] Mezian K., Chang K.V., Zamecnik D., Mezian H., Ozcakar L. (2018). Herniation of Hoffa’s Fat Pad Through the Lateral Retinaculum: Usefulness of Dynamic Ultrasonography to Diagnose a Lateral Knee Mass. Am. J. Phys. Med. Rehabil..

